# Supercomputers Ready for Use as Discovery Machines for Neuroscience

**DOI:** 10.3389/fninf.2012.00026

**Published:** 2012-11-02

**Authors:** Moritz Helias, Susanne Kunkel, Gen Masumoto, Jun Igarashi, Jochen Martin Eppler, Shin Ishii, Tomoki Fukai, Abigail Morrison, Markus Diesmann

**Affiliations:** ^1^Institute of Neuroscience and Medicine (INM-6), Computational and Systems Neuroscience, Jülich Research CentreJülich, Germany; ^2^RIKEN Brain Science InstituteWako, Japan; ^3^Simulation Laboratory Neuroscience – Bernstein Facility for Simulation and Database Technology, Institute for Advanced Simulation, Jülich Aachen Research Alliance, Jülich Research CentreJülich, Germany; ^4^Bernstein Center Freiburg, Albert-Ludwig University of FreiburgFreiburg, Germany; ^5^High-Performance Computing Team, RIKEN Computational Science Research ProgramKobe, Japan; ^6^Laboratory for Neural Circuit Theory, RIKEN Brain Science InstituteWako, Japan; ^7^Integrated Systems Biology Laboratory, Department of Systems Science, Graduate School of Informatics, Kyoto UniversityKyoto, Japan; ^8^Institute of Cognitive Neuroscience, Faculty of Psychology, Ruhr-University BochumBochum, Germany; ^9^Medical Faculty, RWTH Aachen UniversityAachen, Germany

**Keywords:** supercomputer, large-scale simulation, spiking neural networks, parallel computing, computational neuroscience

## Abstract

NEST is a widely used tool to simulate biological spiking neural networks. Here we explain the improvements, guided by a mathematical model of memory consumption, that enable us to exploit for the first time the computational power of the K supercomputer for neuroscience. Multi-threaded components for wiring and simulation combine 8 cores per MPI process to achieve excellent scaling. K is capable of simulating networks corresponding to a brain area with 10^8^ neurons and 10^12^ synapses in the worst case scenario of random connectivity; for larger networks of the brain its hierarchical organization can be exploited to constrain the number of communicating computer nodes. We discuss the limits of the software technology, comparing maximum filling scaling plots for K and the JUGENE BG/P system. The usability of these machines for network simulations has become comparable to running simulations on a single PC. Turn-around times in the range of minutes even for the largest systems enable a quasi interactive working style and render simulations on this scale a practical tool for computational neuroscience.

## 1 Introduction

Supercomputers are employed for different applications arising in the field of neuroscience, such as visualization of neuronal data and simulations of neuronal dynamics (recently reviewed in Lansner and Diesmann, 2012). The human brain exhibits a sparse, recurrently, and specifically connected network of about 10^11^ neurons, each having of the order of 10^4^ synapses to other neurons; its simulation is challenging due to the required memory to represent the structure and the simulation time to solve the dynamics. Such simulations naturally call for the use of supercomputers; machines at the current frontier of processing capability. The neuroinformatics tools employed in this endeavor must be adapted to the computer platforms, even though these systems have typically not been designed with the specific requirements of neuroinformatics applications in mind. The primary objective driving the development of the majority of supercomputer architectures is maximizing floating point performance, rather than providing the large amounts of working memory and high memory bandwidth required by neuronal network simulations. Moreover, the data transfer to and from these machines is often problematic, although specialized parallel I/O solutions do exist (Frings et al., 2009). However, tools cannot be developed solely to capitalize on the properties of supercomputer architectures – first and foremost they must serve the demands generated by the neuroscientific domain.

The choice of the level of abstraction on which neuronal dynamics is represented depends on the scientific question to be investigated. If sub-cellular processes, such as molecular diffusion or the membrane potential propagation along the neurites of a cell are in the focus of the investigation, or suspected to be decisive for the function of the particular system at hand, neurons need to be represented by detailed many-compartment models (Hines, 1984). The simulator NEURON (Hines and Carnevale, 1997) is tailored for this domain of detailed compartmental models, implements efficient solvers for the cable equations (Hines et al., 2008b) and exploits the power of modern supercomputing architectures (Miyamoto et al., 2012) by scalable communication methods (Hines et al., 2008a, 2011; Kumar et al., 2010). In simulators which focus on the investigation of the dynamics of large scale networks (Lansner and Diesmann, 2012), neurons are typically abstracted to single-compartment or few-compartment models that interact by discrete electrical pulses (spikes). The level of description in terms of neurons and synapses, rather than biochemical or biophysical processes within single neurons, is a simplifying assumption that constrains the parameter space of such neuronal network models and entails technical advantages such as low per-object memory usage and relatively simple parallelization of algorithms.

The distributed simulation of spiking networks on parallel computers was exploited early on by the SPLIT simulator (Hammarlund and Ekeberg, 1998; Djurfeldt et al., 2005). This technology enabled pioneering simulations of up to 22 million neurons (Djurfeldt et al., 2008) and is still in use for the production of neuroscientific results (Lundqvist et al., 2010). Available simulation technologies differ in terms of the user interface. While SPLIT (Djurfeldt et al., 2008) provides a C++ library to be linked into the user’s application, the simulators PCSIM (Pecevski et al., 2009) and NEST (Gewaltig and Diesmann, 2007) are controlled by the scripting language Python (Eppler et al., 2009), which has achieved wide acceptance in recent years in the neuroscience community.

In the last decade brain researchers have constructed and simulated models of about one cubic millimeter of brain tissue at the resolution of neurons and their connecting synapses (Amit and Brunel, 1997; Brunel, 2000; Morrison et al., 2007a; Potjans and Diesmann, 2011). This local network model comprises 10^5^ neurons and a total of 1 billion connecting synapses. The predictive power of the model is, however, severely limited as 50% of the inputs to each neuron originate in brain areas outside the local circuit (Stepanyants et al., 2009). Brain functions such as vision involve circuits spanning multiple interconnected areas each comprising on the order of 10^8^ neurons. The visual system of the macaque monkey, for example, has about 5 × 10^8^ neurons, the by far largest area of which (V1) has 2 × 10^8^ neurons (van Essen, 2005; Collinsa et al., 2010). Being able to simulate brain-size networks, the C2 simulator (Ananthanarayanan and Modha, 2007; Ananthanarayanan et al., 2009) implements a similar hybrid scheme of time-driven update of the neuronal dynamics and event-driven update of synapses as the NEST simulator (Morrison et al., 2005). The scalable two step communication scheme used in C2, combining one synchronization point per time step imposed by MPI reduce-scatter with pairs of non-blocking MPI send and receive calls has been well documented (Ananthanarayanan and Modha, 2007). However, other processes and data structures are not described in sufficient detail to be reproducible, for example the extraction of the location of postsynaptic targets at connection time and the structure that stores this information of each unit. The reproducibility of these important technical advances and the usability of the C2 simulator by the neuroscientific community in general are hindered by the unavailability of the simulator and its source code. To partially address this issue, here we investigate in detail the data structures required for brain-scale simulations and their performance. The resulting freely available implementation is a general purpose simulator for arbitrary plastic spiking networks of heterogeneous single or few-compartment neuron and synapse types.

When scaling up a network to 10^8^ neurons on supercomputers with up to 10^5^ compute nodes, success depends critically on how well the data structures are distributed. Obviously, the 10^4^ synapses per neuron dominate the memory consumption. Though connectivity between brain areas is sparse, there are fewer constraints within areas. A general simulation tool needs to be able to simulate networks with arbitrary connectivity. For the memory consumption, random networks present the worst case scenario for two reasons: firstly, there is no redundancy that allows the representation of synaptic connectivity to be compressed. Secondly, communication between the compute nodes is potentially all-to-all. Note that random connectivity is not the worst case for all aspects of simulator performance; it is typically advantageous for load-balancing, as has recently been pointed out (Hines et al., 2011), which also means that it is beneficial for making accurate measurements of the memory consumption. In the case of random connectivity, the distribution of 10^4^ synaptic targets of a neuron over 10^5^ processors results in a highly sparse filling of the data structures representing this connectivity on the target neuron’s machine, and any overhead proportional to the total number of neurons *N* must be kept small.

In previous work we systematically investigated this issue by developing a mathematical model for the memory consumption of the most important data structures. Guided by this model, we presented a design that yielded substantial improvements in memory consumption on massive supercomputers (Kunkel et al., 2012b). In a subsequent work we investigated theoretically (Kunkel et al., 2012a) the effect of the so called “columnar structure” within areas; the probability that two neurons are connected decreases on a typical length scale of a few 100 μm, defining the “cortical column” (Mountcastle, 1997; Sporns et al., 2005). Instantiated for a particular software and computer architecture, the memory model predicts the memory requirement of a planned simulation and hence the size of the required machine. In the present work, we assess the performance of the software and the accuracy of the memory model described in Kunkel et al. (2012b) by simulations carried out on two currently available supercomputer architectures. In Sec. 1, we describe the critical data structures for the distributed representation of synaptic connectivity and extend the model of memory consumption to account for thread-specific data structures in the NEST simulator. In Sec. 2 we employ the model to specify benchmark simulations, investigating whether by the trade-off between memory and computation time, we have now arrived at a practically usable tool for neuroscience. We employ a BlueGene/P architecture by IBM (JUGENE at the Research Centre Jülich, Germany) and the K computer by Fujitsu (RIKEN Advanced Institute for Computational Science in Kobe, Japan). The conceptual and algorithmic work described here is a module in our long-term collaborative project to provide the technology for neural systems simulations (Gewaltig and Diesmann, 2007).

## 2 Materials and Methods

### 2.1 Specification of employed computer architectures

The K computer, located at the Advanced Institute for Computational Science in Kobe, Japan, is a distributed-memory supercomputer system that includes 88,128 CPUs (705,024 cores) and 1.4 PB RAM (Yonezawa et al., 2011). The theoretical performance of the system is 11.28 PFlops. A compute node in the K system is mainly composed of a CPU, memory modules of 16 GB, and a chip for the interconnect of nodes. The CPU architecture is a SPARC64 VIIIfx developed by Fujitsu, Ltd., which has 8 cores with 32 kB data cache each and 6 MB shared L2 cache, operating at a clock frequency of 2 GHz. Theoretical performance per chip is 128 GFlops. Each core has two SIMD units that concurrently execute four double precision floating point multiply and add operations. The compute nodes are connected with the “Tofu” (*to*rus connected *fu*ll connection) interconnect network, a six-dimensional mesh/torus network (Ajima et al., 2009). The bandwidth per link is 5 GB/s. A three-level parallel programming model is available on the K computer: (1) SIMD processing in the core, (2) thread programming in a compute node using OpenMP directives, (3) distributed-memory parallel programming with MPI.

The JUGENE computer was in operation at the Jülich Research Centre, Germany from 2008 to 2012. It is a BlueGene/P distributed-memory supercomputer system developed by IBM that included 73,728 compute nodes (294,912 cores) and 144 TB RAM. Its theoretical performance was 1 PFlops. In the BlueGene/P architecture, each compute node has a 32 bit Power PC 450 CPU, which has 4 cores running at 850 MHz, a shared 4-way SMP L3 cache of 8 MB, and 2 GB of RAM. Each core has a dual floating point unit. The theoretical performance per chip is 13.6 GFlops. The compute nodes are connected with a three-dimensional torus network with a bandwidth per link of 425 MB/s.

### 2.2 Simulation technology

The NEST simulator (Gewaltig and Diesmann, 2007) represents individual single- or few-compartment neuron models as small systems of differential equations which interact by δ-impulses. The technology to simulate networks of these model neurons has been documented in an ongoing series of publications (Rotter and Diesmann, 1999; Morrison et al., 2005, 2007a,b; Plesser et al., 2007; Morrison and Diesmann, 2008; Hanuschkin et al., 2010; Kunkel et al., 2012a,b) and the code is freely available. For a large class of frequently used neuron models, the time evolution of the dynamic equations is essentially linear and can often be integrated exactly (Rotter and Diesmann, 1999). This also holds for many abstracted forms of spike-timing dependent plasticity, including models in which neuromodulators influence synaptic plasticity as a third factor (Morrison et al., 2008; Potjans et al., 2010). Non-linearities are typically concentrated in a single thresholding operation. The NEST simulator implements a hybrid update scheme: time-driven updates in regular intervals propagate the neuronal dynamics and event-driven update of synapses is performed only when the pre-synaptic neuron fires an action potential (Morrison et al., 2005, 2007a,b). Efficient time-driven update schemes have also been developed that enable the exact evolution of the neuronal dynamics, representing the firing times in double precision (Morrison et al., 2007b; Hanuschkin et al., 2010).

In time discrete simulations, for each time step (by default 0.1 ms) the update of a single neuron comprises a propagation step, a modification of initial conditions due to incoming spikes, and the control flow that includes transitions between different internal states a neuron can assume, such as to handle a refractory period after the emission of a spike. For neurons with linear subthreshold dynamics, the propagation step can be represented as an implicit matrix vector multiplication of low dimension (typically <6); for non-linear neuron models a numeric solver is used. For the common case that the immediate effect of incoming spikes on the neuron dynamics is linear (e.g., linear currents or conductances), incoming spikes can be lumped together within a time step in a ring buffer. This enables an efficient representation of short conduction delays on the order of milliseconds, quantized in units of the simulation time step (Morrison et al., 2005). For the simplest models of this class, the number of floating point operations required per neuron and time step can be as low as 2, in addition to the on average 1–10 floating point additions required to accumulate the synaptic input in the ring buffer. Although spike-timing dependent plasticity (STDP) requires only a few extra floating point operations at irregularly spaced time points determined by the spikes of the pre-synaptic neurons (Morrison et al., 2007a, 2008), the synaptic dynamics accounts for a comparable amount of additional floating point operations, because synapses outnumber neurons by a factor of 10^4^. The neurons of the network are evenly distributed over the compute nodes in a round-robin fashion and communication between machines is performed by collective MPI functions (Eppler et al., 2007).

The compute nodes in contemporary supercomputers, like JUGENE and K, contain multi-core processors (see Sec. 1); the trend toward ever greater numbers of cores is further manifested in the new BlueGene/Q architecture with 16 cores per node, each capable of running 4 hardware threads. These architectures feature a multi-level parallel programming model, each level potentially operating at different granularity. The coarsest level is provided by the process based distribution, using MPI for inter-process communication (Message Passing Interface; Pacheco, 1997). Within each process, the next finer level is covered by threads, which can be forked and joined in a flexible manner with OpenMP enabled compilers (Board, 2008). The finest level is provided by streaming instructions that make use of concurrently operating floating point units within each core. The code used in this work combines distribution by MPI, starting one process per compute node and utilizes multi-threaded OpenMP-based software components within each process during the setup and simulation phase. The use of threads instead of one MPI process per core is essential, because each MPI process entails an additional memory overhead due to replicated data structures and the process management. Moreover, the communication load and memory consumption caused by the currently employed collective data exchange scheme (Morrison et al., 2005; Plesser et al., 2007) increases with the number of MPI processes.

## 3 Results

### 3.1 Adaptations of the memory usage model to threading

In Kunkel et al. (2012b), we presented a model that enables the analysis of the memory usage of a neuronal network simulator. The model expresses the memory consumption of each MPI process as a function of the total number of neurons *N*, the number of incoming connections per neuron *K*, and the number of MPI processes *M*. Here, we instantiate the model terms and parameters for the simulation software NEST (revision 9630) in order to obtain reliable predictions of the maximum size of a randomly connected neuronal network that fits on a specific supercomputing architecture without saturating the available memory resources. We extend the original formulation of the memory usage model to account for threading, which results in

$$\begin{array}{rcll} ℳ\left(M,T,N,K\right) & ={ℳ}_{0}\left(M\right)+{ℳ}_{\text{n}}\left(M,N\right) & & \\ & \;+{ℳ}_{\text{c}}\left(M,T,N,K\right) & & \text{(1)} \end{array}$$

where *T* is the number of threads per MPI process. The three components ℳ_0_ (*M*), ℳ_n_ (*M*, *N*), and ℳ_c_ (*M*, *T*, *N*, *K*) denote the base memory consumption of the simulator including the contributions of MPI, the memory usage of neurons and neuronal infrastructure, and the memory usage of connections and connection infrastructure, respectively. The first term ℳ_0_ (*M*) is the memory consumption after startup due to code and data structures prior to the creation of any neurons or synapses. We obtain this value by measurement, as it depends on the computer platform, the compiler, the employed libraries and the linking procedure (static/dynamic). The second term is the memory consumption of neurons. It is dominated by the storage of the state variables, which is typically around 1000B per neuron. In addition it contains the contribution from a sparse table (Silverstein, 2005) needed to check for local existence of a neuron on a given compute node and the overhead caused by the vector storing the local neurons. These contributions to the memory consumption are rather minor in the regime of up to 32,768 compute nodes considered here. In order to account for threads as they are implemented in NEST, only the third model term needs adaptation while the first and the second model term remain unaltered.

**Figure 1:**
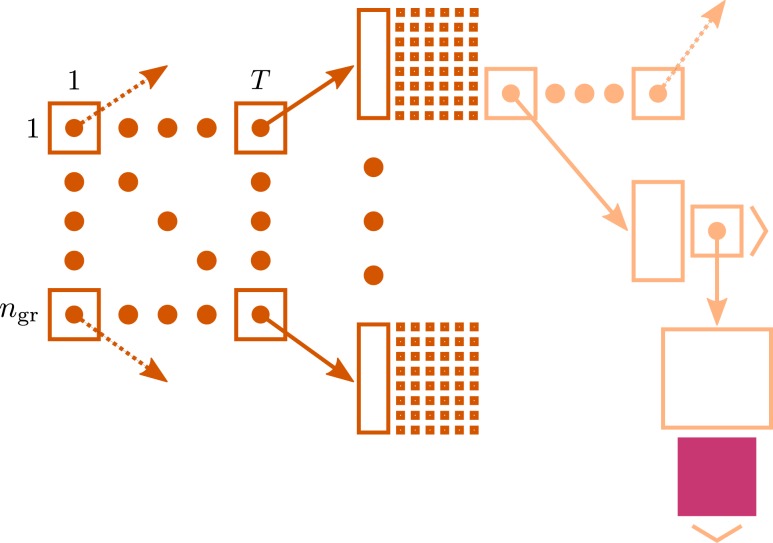
**Connection infrastructure in NEST optimized for supercomputers for the case that a simulation is run with *T* threads**. The *n*_gr_ × *T* matrix of dark orange squares illustrates the vector of length *T* which provides thread-specific access to sparse tables (Silverstein, 2005), where each sparse table maintains *n*_gr_ sparse groups. Vertical dark orange rectangles and tiny squares indicate the per-group overhead of the sparse table. For simplicity, the figure shows only once the additional infrastructure which is required for each neuron with local connections (light orange). The filled pink square illustrates a locally stored connection object. Figure adapted from Kunkel et al. (2012b).

Figure 1 illustrates the connection infrastructure of NEST that is required on each compute node for the case that a simulation is run with *T* threads. On the highest level a vector of dimension *T* holds a sparse table (Silverstein, 2005) for each thread. Connections are represented on the same MPI process, and further, on the same local thread *t* ∈ [1, …, *T*] as their postsynaptic targets. For each neuron *j* ∈ [1, …, *N*] in the network, the sparse table for a specific local thread *t* ∈ [1, …, *T*] stores the information about whether neuron *j* has any targets on thread *t*. To this end, the index space 1, …, *N* of all neurons is equally partitioned into *n*_gr_ subgroups (here 48 entries). For each entry in a group, 1 bit is stored in the bit-field (tiny squares in Figure 1) indicating the existence of a target neuron for the specific presynaptic neuron *j*. If neuron *j* has at least one target, the sparse table stores a pointer to a vector, which enables different synapse types (e.g., representing plasticity rules) to be distinguished, such that connections of the same type can be stored in a homogeneous vector. For a detailed description of the fundamental data structures in NEST and how these can be mapped to the model terms please see Kunkel et al. (2012b).

On each thread, this infrastructure causes an overhead of $m_{\text{c}}^{0}$ per neuron. We denote such a linear dependence on the total number *N* of neurons *serial overhead*, indicating that this contribution does not benefit from distributing the *N* neurons over *M* parallel machines. Additionally each neuron in the network causes an overhead of $m_{\text{c}}^{+}$ if it has locally stored target synapses and $m_{\text{c}}^{\emptyset }$ without, such that

(2) $$\begin{array}{rc} {ℳ}_{\text{c}}\left(M,T,N,K\right) & =TNm_{\text{c}}^{0}+TN_{\text{c}}^{\emptyset }m_{\text{c}}^{\emptyset }\\ & \;+T\left(N-N_{\text{c}}^{\emptyset }\right)m_{\text{c}}^{+}\\ & \;+K_{M}m_{\text{c}} \end{array}$$

gives the memory usage of connection infrastructure and connection objects per MPI process, where *m*_c_ is the memory consumed by one connection object. Assuming neurons and their incoming connections are distributed evenly across processes, *N_M_* = *N*/*M* neurons and *K_M_* = *N_M_K* synapses are represented on each MPI process, and *N_T_* = *N_M_*/*T* neurons and *K_T_* = *N_T_K* synapses are represented on each thread. Due to random connectivity the probability that a neuron has no local targets on a specific thread is *p*_∅_ = (1 − 1/*N*)*^K_T_^* which results in an expected number of $N_{\text{c}}^{\emptyset }=p_{\emptyset }N$ neurons without local targets on each thread. Please see Kunkel et al. (2012b) for a detailed description of the memory usage model and a comprehensive demonstration of how the model can be applied to predict the memory consumption of a simulation software. The parameters used for the predictions in Figure 4 are displayed in Table 1. The value for the memory consumed by a single connection object *m*_c_ is based on a representation of the synaptic parameters and weight in double precision, as this allows for most generality and represents the underlying mathematical model most accurately. However, it should be noted that if a lesser precision (for example using the float data type) is adequate for the scientific question at hand, a lighter weight synapse can of course be implemented without any change to the framework.

**Table 1:** **Parameters used to instantiate the memory model (2) on K and JUGENE**.

Parameter	*m*_c_ (Byte)	$m_{\text{c}}^{+}$ (Byte)	$m_{\text{c}}^{0}$ (Bit)	$m_{\text{c}}^{\emptyset }$ (Byte)	*K*
Value	48	136	2.67	0	11,250

*Consumptions per element $\left(m_{x}^{y}\right)$ were obtained by fitting the memory consumption in simulations on JUGENE varying network parameters independently, as described in Kunkel et al. (2012b)*.

### 3.2 Performance of NEST on K and JUGENE

We use a recurrent random network of current-based integrate-and-fire model neurons with spike-timing dependent plasticity in the connections from excitatory to excitatory neurons as a benchmark simulation. All parameter values for the neuronal dynamics and the details of the employed models are taken from Morrison et al. (2007a). The parameters of the spike-timing dependent plasticity rule are τ_+_ = 15 ms, τ_−_ = 30 ms, λ = 0.1, μ = 0.4, and α = 0.0513. In order to perform scaling experiments, we varied the number of neurons in the network, keeping the number of incoming synapses per neuron constant (each neuron receives *K_E_* = 9000 excitatory and *K_I_* = 2250 inhibitory incoming connections that are drawn randomly from the respective pools of source neurons). Constant numbers of inputs ensure that networks of different sizes are in comparable dynamical states. In particular, the firing rate averaged over neurons in the network is unaffected by network size $\left(\nu ≃6.9\frac{1}{\text{s}}\right)$.

Our benchmark model contains a Poisson source to model external input to the network (Morrison et al., 2007a). These sources are stochastic by definition. Moreover, the connectivity of the network is generated randomly. However, the random numbers required to realize the connectivity and the Poisson spike trains are drawn such that identical sequences are produced when rerunning the same simulation. This is crucial not only to be able to reproduce scientific results, but also to have a means of testing different implementations against each other during software development, in particular to benchmark performance improvements. The mechanisms to achieve this reproducibility even across different numbers of MPI processes are described in Morrison et al. (2005), Plesser et al. (2007). The key ingredients of the implementation are the use of thread-local random number generators, initialized with the same seed at the beginning of each run and the parallelization of connection setup routines that ensure that the same numbers of random variates are drawn from each thread-local generator irrespective of the number of processes.

We use identical versions of the NEST software (revision 9630) on both supercomputing architectures. The code incorporates the optimizations for large supercomputers as described in Kunkel et al. (2012b) and will be available in the next release of NEST and accessible via the K software site. The code was compiled with the respective proprietary C++ compilers [XL C/C++ V9.0 for BlueGene/P on JUGENE and on K the Fujitsu C/C++ compiler, version 1.2.0 (build February 27 2012) of the K development phase 1.2.0-04]. No code changes were introduced to exploit particular features of the proprietary compilers; the same code can be compiled with the standard compiler g++ (version 4.4).

**Figure 2:**
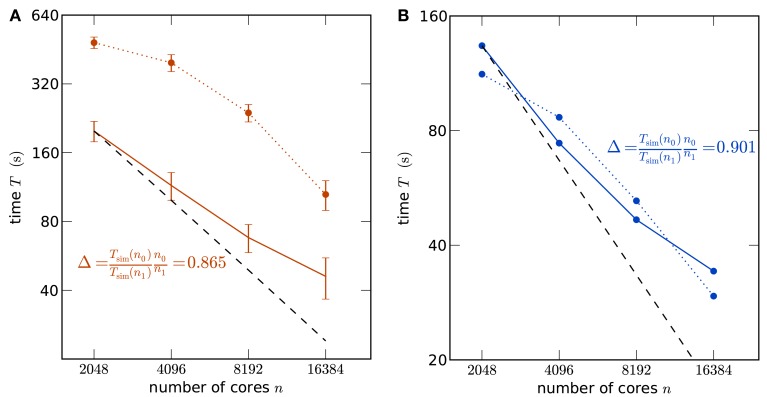
**Strong scaling of the NEST simulator**. **(A)** A network of *N* = 3,502,080 neurons is simulated on K for 1 s of the biological system. Optimal linear scaling is shown by the dashed line. At 2048 cores the network consumes all available memory, causing the highest possible workload per core for the given network size. The scaling of the simulation time (solid curve) between 2048 and 4096 cores has a slope slightly below linear scaling (0.865). The error bars denote the standard error of the mean from 5 repetitions of identical simulations (same random numbers). The setup time (allocation and creation of neuron objects and synapses) is shown by the dotted curve. **(B)** Strong scaling for a network of *N* = 1,126,400 neurons on JUGENE. At 2048 cores the network consumes all available memory. Same symbol code used as in **(A)**.

In order to assess whether the simulation code makes good use of the parallel architecture of the supercomputers, we show a strong scaling of the simulation time in Figure 2A using K and in Figure 2B using JUGENE, keeping the problem size (number of neurons) constant while increasing the number of used processor cores. In particular the communication at regular intervals between different machines required to deliver the spikes (point events in time) to the target neurons imposes synchronization points for the threads and the MPI processes, possibly limiting the scalability of the application. For a fixed size of the network of about 3.5 × 10^6^ neurons, the strong scaling experiment on the K machine reveals the high degree of parallelism of the hybrid code, resulting in the excellent scaling shown in Figure 2A. A high slope of 0.865 can be observed for the simulation time near the point of maximum filling (here at 2048 cores), where the load per processor is highest. Changing the seeds of the random number generators results in different realizations of the random networks. This has a negligible effect on the firing rates in the network and therefore on the simulation time. The largest source of fluctuations in the runtime of these simulations are load differences on the compute nodes caused by other users. To quantify these fluctuations we performed for each number of nodes 5 identical simulations for the strong scaling on K. The standard error of the mean over the 5 runs is shown as error bars in Figure 2A. Note that this error increases for larger sizes of the machine, as the communication time becomes dominant. Figure 2B shows the corresponding strong scaling on JUGENE. The size of the maximum filling network at 2048 cores is about 1 × 10^6^ here, due to the smaller amount of working memory per CPU compared to K. At this point of highest load per processor, the slope reaches 0.90.

For such large networks, not only the simulation time needs to be taken into account, but also the setup of the network may consume a considerable amount of time. In the current implementation, the wiring process is therefore also performed in parallel on two levels, firstly on the coarse grained level of MPI processes and secondly employing finer grained parallelism implemented with OpenMP directives. As the synaptic information is exclusively stored on the machine that harbors the target neuron in a thread-specific structure, as shown in Figure 1, both levels of parallelization are implemented in a natural way: each MPI process and thread establishes and stores the incoming connections for the neurons that are local to that process and thread. The second level of parallelization using one OpenMP thread per available core (4 on JUGENE and 8 on K) is possible because the threads work independently on disjoint parts of the connection infrastructure (see Figure 1). Figures 2A,B show the scaling of the time required for network setup. The absolute value is below 10 min at the highest load per processor. As a production run for the network considered here is typically longer than 1 s of biological time (Morrison et al., 2007a; Kunkel et al., 2011), the efficiency achieved by the network setup is sufficient for practical applications. In contrast to the simulation time, the speedup for the setup time increases with decreasing load per processor and almost reaches optimal linear scaling at the point at which the machine is 4 times larger (8192 cores) than the minimal required size (2048), as seen in Figures 2A,B. Near the point of maximum filling, connection setup is less effective. This hints that the memory allocation for the synaptic infrastructure may be dominating the setup time.

Strong scaling for simulation and network setup are important measures to assess the percentage of parallelism achieved by the application, but this measure is less informative for the typical use of the simulation tool by a neuroscientist. Given a neuroscientific question to be investigated, the number of neurons N is determined by the chosen model of the biological system. The researcher needs to determine the size of the machine required to address this question by simulation. It is desirable to determine the minimum size of the machine in terms of number of CPUs and working memory that is sufficient in order to keep the energy consumption small and because the effort spent on computation time grant applications typically increases with the size of the machine asked for. Moreover, the shared use of high performance computing resources by a large community of users requires thoughtful behavior of the individual. Using the smallest possible portion of the machine for a given task causes faster scheduling and thus often leads to shortest return times, as the startup plus the queuing time contribute considerably to the turn-around times. In the case of spiking network simulations, the feasibility of a particular simulation is determined by memory constraints rather than by the required performance of the machine. In the following we therefore study a “maximum filling scaling”: for a given problem size (number *N* of neurons) we use the smallest portion of the computer that has sufficient memory to fit the requirements of the simulation.

**Figure 3:**
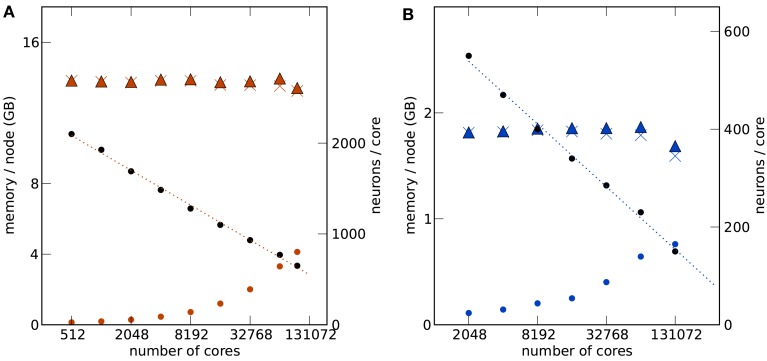
**Maximum filling scaling**. The network size (number of neurons) is chosen so that the simulation consumes all available memory. The affordable number of neurons per core (black round symbols) decreases with increasing number of cores to keep the total memory consumption close to the usable maximum. The dotted lines give linear fits to the data. The memory consumption at different stages of the simulation (colored round symbols: after allocating the neurons, colored crosses: after establishing the synapses, colored triangles: after running the simulation) show the memory consumption at different stages of the simulation. The largest contribution is due to the synapses. All data are represented using log-linear axes. **(A)** K computer with 13.85 GB (nominal 16 GB) memory per compute node. **(B)** JUGENE computer with 1.84 GB (nominal 2 GB) memory per compute node. Note that the last point at 131,072 cores is slightly below the maximum memory usage, as the bisectioning method we used to empirically determine the largest possible number of neurons per core did not converge before the end of our access period to K.

**Figure 4:**
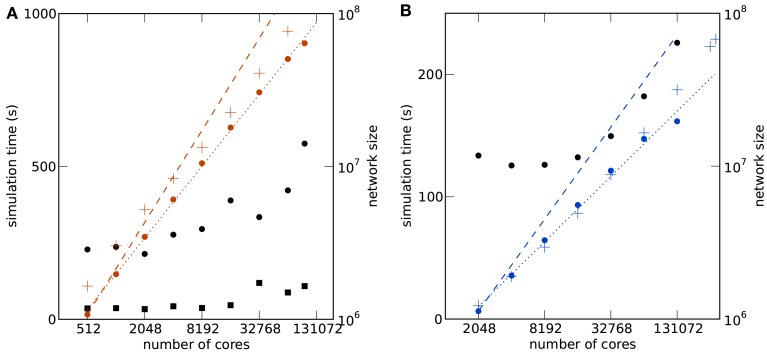
**Maximum filling scaling**. Simulation time required for 1 s of biological time is shown as black round symbols for a firing rate per neuron of $6.9\frac{1}{s}$ and as black square symbols for $1\frac{1}{s}$. Total size of the network (i.e., number of neurons) is shown as a function of the number of cores (colored round symbols). The theoretical prediction (1) of the maximum possible network size is shown as colored crosses. Optimal linear scaling shown as dashed lines; the dotted lines give linear fits to the data. **(A)** K computer; the estimated slope of the maximum network size is 0.780. **(B)** JUGENE computer; the estimated slope is 0.708.

Figures 3 and 4 show such a maximum filling scaling of NEST on K and JUGENE. The memory consumption of neurons increases with the number of cores, because of memory overhead that is serial in *N*. Each instance of NEST has a sparse table for the *total* number of neurons, which is needed to determine whether a neuron is locally represented (Kunkel et al., 2012b). Similarly, the sparse table in the connection infrastructure (see Figure 1), needs to store for each neuron 1, …, *N* in the network whether it has a target on the local machine. As both structures grow proportional to *N*, they constitute a serial memory overhead. As a result, in Figures 3A,B the number of neurons per core needs to decrease with increasing network size in order to remain within the memory constraints on each compute node. In the log-linear plot the number of neurons per core decreases in a linear fashion, demonstrating an approximately logarithmic dependence on the number of cores. The linear extrapolation has an intercept with the *x*-axis at a certain number of cores exposing the limits of the current implementation. Correspondingly, the *total* number of neurons as a function of the machine size, shown in Figures 3A,B, increases slightly sub-linearly.

The memory model developed in Kunkel et al. (2012b) and adapted to the hybrid simulation scheme in Sec. 1 is shown by the cross symbols in Figure 4. The agreement for the JUGENE computer is good and approximately reproduces the sub-linear slope. At small numbers of cores the memory consumption is slightly overestimated, at larger machine size the theory is below the measured value. A possible source of error is the memory management system of the operating system. The memory overhead involved in managing dynamic memory allocation within the kernel is not part of our model. These structures, however, are expected to reach considerable sizes, as during network setup a large number of small allocations is performed for small synapse objects of ∼100 bytes each, filling the complete memory available. Future implementations will need to face this issue, for example by employing more effective pool allocation strategies (Stroustrup, 1997). The deviations of the model from the measured memory consumption for the K computer is larger than for JUGENE (see Figure 4), but the slopes of theoretical and estimated curves approximately agree. A possible reason for the deviations is the method of the memory consumption measurement: while we used a dedicated library function of the IBM C++ compiler (Kernel_GetMemorySize) on JUGENE, on the K computer we read out the entry for virtual memory in the /proc file system. For the latter, we observed a non-monotonic dependence on, e.g., the number of allocated synapse objects not expected theoretically. As we rely on these measurements in order to determine the parameters of the memory model (see Sec. 1), deviations can be expected.

With increasing machine size the simulation time shown for JUGENE in Figure 4B first decreases and then increases. The decrease is due to the reduced workload (neurons per core, see Figures 3A,B). As we are using collective MPI communication, the communication time increases with the machine size. This explains the subsequent increase of the simulation time at higher numbers of cores. On K the simulation time and the slope of the simulation time predominantly increase with machine size (Figure 4A). The fluctuating behavior of the runtime on the K computer is mostly due to fluctuations of the communication time, presumably caused by other users who are running communication or I/O-intense applications at the same time. Different configurations of the Tofu communication topology (see Sec. 1) also have an effect on the simulation time: an allocated number of *M* compute nodes can be logically arranged as a topological torus with dimensions *k* × *l* × *m* = *M*. In this work we did not explicitly specify the topology *k* × *l* × *m*, but simply specified the total number of nodes *M*. Rerunning the same simulation in Figure 4 with different topologies showed a notable effect on the runtime. For an application like NEST that relies on collective all-to-all communication, the naive expectation is that a cube like configuration $\left(k≃l≃m≃M^{\frac{1}{3}}\right)$ should result in shortest communication latencies. However, this turned out to be false. As the K software environment matures, further investigations into the optimal configuration can be carried out.

The simulation time on JUGENE for 1 s of biological time is below 240 s even for a network of 2.0 × 10^7^ neurons with an average rate per neuron of $6.9\frac{1}{s}$. On K the largest network of 6.4 × 10^7^ neurons executes in less than 600 s for the same biological time and rate. Decreasing the firing rate in the network to $1.08\frac{1}{s}$ by reducing the external Poisson drive to each cell reduces the runtime to below 120 s. The memory consumption was only marginally affected (data not shown). The short return times allow for a quasi interactive working style with the model for short simulations of a few seconds of biological time.

**Figure 5:**
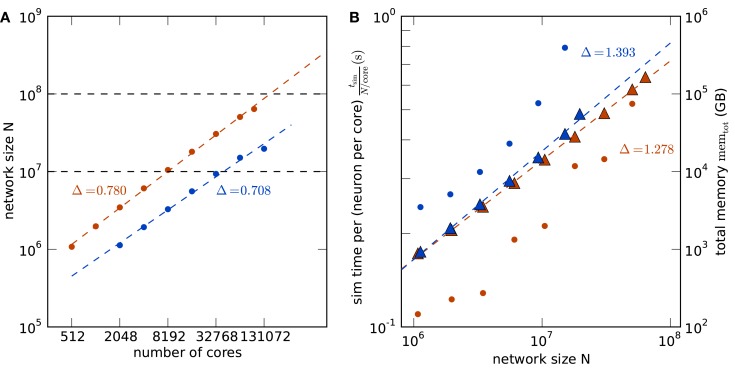
**Comparison of K (red) and JUGENE (blue)**. **(A)** Maximum possible network size as a function of the number of cores (same data as in Figures 4A,B). The dashed lines are determined by a linear fit to the data points. The lines end at the full size of the respective machine (294,912 cores for JUGENE, 705,024 cores for K). The dashed horizontal lines at 10^7^ and 10^8^ neurons are given for visual guidance. **(B)** Memory consumption and runtime. Simulation time normalized by the workload *N*/core (number of neurons simulated per core) of the processor as a function of the simulated network size (round symbols). Total memory consumption mem_tot_ as a function of the network size *N* (triangles). The dashed lines are linear fits to the data with slopes indicated by the symbols Δ.

Figure 5 shows the comparison of the two supercomputers. The maximum size of the network for a given machine size is shown in Figure 5A. Due to the larger memory per core of K compared to JUGENE (2 vs. 0.5 GB), the maximum possible network size on K exceeds that on JUGENE; at 16,384 cores the factor is about 3.24. The slightly higher slope on K compared to JUGENE indicates that this ratio increases toward larger machine sizes. In the optimal case without any serial overhead in the representation of the network, one would expect a factor of 4 corresponding to the relative size of the total available working memory. A similar observation can be made from the total memory consumption as a function of the network size, shown in Figure 5B. The slope of the linear fit is above the optimal linear scaling (slope 1) and the memory increase on the K computer is slightly less than on JUGENE. With the current technology, the absolute size of 10^8^ neurons can be reached on the K computer, but not on JUGENE.

Normalizing the simulation time by the workload per core exhibits an increase with the number of cores in Figure 5B, caused by the collective communication scheme. As each spike produced within the network needs to be communicated to all other processors irrespective of targets existing on that machine, the communication load increases with the number of cores. Additionally, the number of spikes arriving at a given machine grows almost in proportion to the number of cores. So as the network size increases, so does the number of arriving spikes, each of which needing to be checked for whether it has local targets, and so too increases the proportion of spikes which have no such local targets and must be discarded. The resulting increase in run time on the K computer is less steep than on JUGENE, because K has double the number of cores per CPU than JUGENE and thus requires half the number of MPI processes for a given number of cores.

The comparison of the execution time per workload, shown in Figure 5B, obviously reflects the different clock speeds of K (2 GHz) and JUGENE (850 MHz). Note that the peak performance of a single CPU in K measured in FLOPS is almost a factor 10 larger than that of JUGENE (see Sec. 1, K: 128 GFlops, JUGENE: 13.6 GFlops). The peak performance of K can be achieved by streaming instructions (single instruction multiple data, SIMD) utilizing both concurrently working floating point units in each core. Here, however, we used the same C++ code on both machines that does not employ this technique, so not making use of the full floating point performance of K. Moreover, the benchmark considered in this contribution uses rather lightweight computations per neuron, where each simulation time step comprises only a few floating point multiplications and additions per neuron. However, the delivery of spikes to randomly drawn target neurons causes frequent and random memory access. Therefore, for our application the floating point performance of the CPU is less relevant than clock speed, cache efficiency, and memory bandwidth.

In order to measure the performance of NEST on K, we employed the profiling tools fpcoll and fprofx that are part of the Fujitsu development kit. We simulated 18,420,000 neurons on a subset of 98,240 cores (12,280 nodes). We obtained the number of floating point operations per second (MFLOPS) and instructions per second (MIPS) as shown in Table 2. Note that the total number of floating point instructions in the simulation code is very low in our benchmark, as we are using a leaky integrate-and-fire neuron with only three state variables and the exact integration method (Rotter and Diesmann, 1999). The floating point performance is thus expected to be low. For a program without floating point operations the measure is zero. The MIPS performance is good, especially considering that the application heavily relies on random memory access.

**Table 2:** **Performance measures of the NEST simulator for 98,240 cores simulating 18,420,000 neurons on the K computer obtained with the fpcoll/fpcollx profiling tool**.

	Per core	98,240 Cores
MFLOPS	13.9547	151775.3847
MFLOPS/PEAK (%)	0.0872	0.0772
MIPS	670.4384	8987312.8521
MIPS/PEAK (%)	8.3805	9.1483

## 4 Discussion

NEST is an openly available tool to routinely simulate networks of spiking neurons of functionally relevant sizes on HPC facilities; its use is also taught in the major advanced computational neuroscience summer schools. The simulation framework as used in this contribution does not sacrifice any generality for efficiency. In particular, the description of the network model is entirely formulated in NEST’s proprietary interpreter language (SLI), not requiring any custom changes on the level of the C++ source code of the simulator. The improved connection infrastructure as presented in Kunkel et al. (2012b) and its adaptation to multiple threads introduced in Sec. 1 allows different synapse models to be represented in the same simulation. This is a crucial prerequisite to investigate synaptic plasticity in recurrent networks, the biological substrate hypothesized to underlie system level learning. Moreover, the same code that we used on supercomputers here also runs on small machines, like laptops, without any penalty in performance.

The improvements to NEST (documented in Kunkel et al., 2012b) put networks of 10^8^ neurons within reach, utilizing just above 12,288 compute nodes of the K supercomputer (<14% of the total machine). This number of neurons is a critical point at which the largest areas of the visual system in the primate brain can be represented at full cellular resolution. Integrating the simulation of microscopic dynamics into the bigger picture of behavior and learning on the systems level ultimately requires simulations at this scale and beyond.

The simulator NEURON implements advanced schemes of communication that allow a partial overlap of the computation and the communication phase (Hines et al., 2011). The network sizes considered in that work at a comparable number of 10,000 synapses per neuron on 131,072 cores reach up to 4 × 10^6^ simple artificial neurons on a BlueGene/P architecture. The largest network in our simulations at this machine size is 19.6 × 10^6^ neurons, around 5 times larger (see Figure 4B). Note, however, that the network sizes in Hines et al. (2011) were not explicitly stated to be the maximum possible number of neurons and in our benchmark the connections implement general STDP dynamics (see Kunkel et al., 2012b, Sec. 2.3).

Using detailed compartmental models instead of point neurons, a simulation of 10,000 neurons of the Blue Brain project’s cortical column model was performed on 1024 CPUs (512 MB per CPU) on a BlueGene/L (cf. Figure 7 in Hines et al., 2008b). The neuroscientist may ask the question: what size of network can be simulated with these computational resources, if all the 10,000 synapses of a neuron are represented and the detailed neuron model is reduced to a point neuron, trading accuracy on the description of the single neuron dynamics for accuracy in the description of network structure? In our Figure 2B we simulate around 1.1 million point neurons on 2048 cores (512 MB per cores) on a BlueGene/P, thus we estimate that slightly over 550,000 neurons would fit on 1024 cores.

Comparing the simulation time for the networks, both firing at roughly $7\frac{1}{s}$ for 1 biological second, yields around 1300 s for the Blue Brain columnar model of 10,000 cells on 2048 cores of BG/L and 133 s for 1,126,400 cells in the NEST simulation on 2048 cores of BG/P. Normalized by the number of neurons, this is 1.3 × 10^−1^ s/neuron for the detailed neuron model and 1.2 × 10^−4^ s/neuron for the point neuron simulation at the same number of cores. These numbers are only very rough estimates, not taking into account the different architectures, cache sizes, and clock speeds (BG/L 700 MHz, BG/P 850 MHz).

The C2 simulator (Ananthanarayanan and Modha, 2007; Ananthanarayanan et al., 2009) is another simulation code dedicated to simple point neuron models. The largest network so far simulated with the specifically written code of C2 was 1.6 × 10^9^ neurons with 5485 synapses each on 147,456 cores of BlueGene/P (144 TB total memory). NEST reaches a maximal network size of 2.0 × 10^7^ neurons with 11,250 synapses on 131,072 cores of BlueGene/P (70 TB total memory). These are around 57.3 synapses per KB in C2 compared to 3.3 synapses per KB in NEST. The larger memory consumption of NEST is due to its generality. In particular, NEST enables the user to combine different synaptic dynamics in the same simulation. This entails that the connection framework consists of flexible data structures capable of storing and distinguishing between heterogeneous synapses types (shown in Figure 1). Moreover, we represented all quantities in double precision, whereas the 16 bytes per synapse of C2 (Ananthanarayanan and Modha, 2007) is presumably achieved using single precision floating point numbers. Comparing the simulation times for the same cases mentioned above, C2 is 643 times slower than real time per Hertz firing rate. Since the synapses cause most of the computation, we normalize the runtime by the workload, the synapses per core, which yields a simulation time per firing rate and workload of $643\;\text{s/}\left(1\frac{1}{s}\;1.6\times 10^{9}\;5485∕147,456\right)=1.1\times 10^{-5}\;\text{s/}\frac{1}{s}$ for C2. For NEST this calculation yields an only slightly larger number of $225\;\text{s/}\left(7.6\frac{1}{s}\;2\times 10^{7}\;11,250∕131,072\right)=1.7\times 10^{-5}\;s∕\frac{1}{s},$ so generality does not compromise performance here.

The two key parameters defining the required computational resources for a given scientific question from the point of view of the neuroscientist are the size of the network and the length of the simulation time. However, in order to investigate this model on a supercomputer, these parameters must be transformed into estimates for the necessary number of cores and the wall clock time. These estimates are not only important for the efficient, sensible, and democratic use of high performance utilities, but are also valuable for potential users planning a computation time grant application, especially for the upcoming open calls for K. The availability of such a model is beneficial over determining computation resources by trial and error, wasting precious computation resources and time. Often such a theoretical prediction is the sole option, in the case that the desired computer resource is accessible only after a successful grant application procedure, which typically asks for accurate estimates of resources in the first place.

By applying our model of memory consumption to a real world simulation scenario, we have shown that this mathematical tool provides reliable estimates for the required number of compute nodes. The necessary machine size is predominantly determined by the number of neurons and their connectivity. This fact is evident from Figure 4, showing that the memory model only considering the contribution of the data structures representing neurons and synapses accounts well for the memory consumption. The firing rate in the network has only a small impact on the required memory. The dominant contribution to the memory load due to spiking arises from the buffers required to store the spike events. These events are communicated in regular intervals determined by the global minimal synaptic conduction delay min(*d*) used in the simulation (Morrison et al., 2005). In our benchmarks this delay is on the order of min(*d*) = 1.5 ms. Given the neurons in the network fire on average with rate $\nu =6.9\frac{1}{s}$ and there are at most *N_T_* = 2000 neurons per core (cf. Figure 3), each core produces on average *N_T_* min(*d*) = 20.7 events per communication step. With collective MPI communication these events are sent to all participating nodes. For the largest employed machine size of 98,394 cores on K in Figure 4 this leads to around 2 million events per communication step. These events need to be stored in a buffer on each node, taking up 4 bytes per global id of the sending neuron plus a small amount of overhead (see Morrison et al., 2005 for the details of the implementation), in total leading to 8.1 MB buffer size on each node. This amounts to only about 0.05% of the working memory available on the node.

The impact of the firing rate on the run time is more pronounced. The profiling analysis of the simulations on the K computer have shown that for machine sizes up 12,288 compute nodes, most of the simulation time is spent in the update function to calculate the synaptic weight changes due to STDP. This function is called at each occurrence of a spike of the presynaptic neuron, so it clearly depends linearly on the firing rate. Therefore, in order to generate similarly reliable estimates for the wall clock time required, it would be advantageous to develop a model of runtime to supplement our model of memory consumption.

Rather than being a purely technical capability demonstration with a code specifically tuned for one particular machine, we show that the usability of supercomputers for the simulation of networks of simple model neurons has now increased to a level comparable to the use of single PCs; the same freely available software that runs on a laptop can be compiled and run on K and JUGENE, executing the same simulation scripts. This enables an unprecedented advantage in computational neuroscience: a researcher can perform much of the work to develop even a very large scale computational model on easily accessed hardware of modest dimensions. When this preliminary work is complete, the model can be scaled up to supercomputers without conversion issues.

Note that this paper focuses on the technical aspects of running such simulations on a supercomputer, rather than the demanding task of defining network models on the brain-scale, which is hindered both by the limited availability of suitable experimental data to constrain the simulation and by the difficulty of extracting the required measures from such data. Addressing these issues remains an important challenge for neuroscience; here we are simply showing that simulator and supercomputer technology have now developed to the point that the actual process of setting up and simulating the network is practical. Within the realm of neuroinformatics there are additional important problems. Finding the right level of abstraction and defining ontologies to describe neuronal network simulations in a reproducible manner is not at all trivial, although promising steps in this direction have been made. On the technical side, common interfaces like PyNN have been defined and implemented that enable the researcher to define neuronal network simulations independent of the employed simulator (Davison et al., 2009). NeuroML is a model definition languages that is suitable to describe neuronal network models, providing a description of the underlying physiology (Gleeson et al., 2010). NineML is targeted at the simulator independent description of neuron and synapse models as well as connection routines that may then provide the primitives to define network models in NeuroML (Raikov et al., 2011). These efforts are crucial to facilitate collaborations and sharing of models within the community.

The current work has identified limits of the present implementation mainly resulting from the memory overhead of the connection framework. Synapses are stored on the machine, where the target neuron resides and the representation of each non-empty list of these local synapses of a given source neuron comes with a certain overhead $m_{\text{c}}^{+}$ (see Sec. 1). There are as many lists as neurons in the network. Thus in a weak scaling, the number of lists increases with network size, while the length of each list decreases accordingly. Consequently, the effective memory consumption per synapse grows. Moreover, any structure growing in proportion to the total number of neurons, like the mentioned sparse tables represents a serial memory overhead that ultimately prevents further scaling. Future work needs to address this issue, investigate optimized distributed storage strategies for networks at the 10^9^ neuron scale and also has to assess the feasibility of collective communication or alternative communication schemes on the full number of nodes of today’s supercomputers. Preparing the simulation technology for brain-scale networks is the basis for further neuroscientific research employing supercomputers and enables us to better assess the resources required for whole-brain simulations.

## Conflict of Interest Statement

The authors declare that the research was conducted in the absence of any commercial or financial relationships that could be construed as a potential conflict of interest.
